# Chicago Medical Society, Meeting of January 4th

**Published:** 1886-02

**Authors:** 


CHICAGO MEDICAL SOCIETY.

*Stated Meeting January 4th, 1886.*—The President, C. T. PARKES, M. D., in the chair.

The first paper read was entitled *The Effects of Cocaine on the Central Nervous System*, by PROFESSOR D. R. BROWER.

He said we have recovered from the primary effects of the brilliant discovery of Dr. Carl Koller, that sixteen months ago electrified the medical world, and we can now reason together calmly and dispassionately about this powerful therapeutic agent.

He said he had been using in private and hospital practice preparation of the coca leaf for about six years, and for about one year past the alkaloid cocaine, and had reached certain conclusions as to its beneficial and its baneful effects on the central nervous system, that he proposed to present for discussion in his paper. He said beneficial and baneful effects, for it is as powerful for evil as it is for good.

First. *Its Effect upon the Brain.*—In small doses, that is, three or four drachms of the infusion, or one-half to one grain of the alkaloid, it is the most certain and agreeable of all cerebral stimulants. It increases the frequency of the pulse and respiration, and elevates the body temperature. It gives a sense of well-being, a freedom from care, and a pleasant mental exaltation. The first effect of the drug is upon the cerebrum, then upon the medulla oblongata, the sense of mental exhilaration preceding the stimulation of respiration and circulation. In small doses it also stimulates the spinal cord, producing a desire for muscular activity, and increasing activity of reflexes.

The effect upon the spinal cord, according to the experiments of Dr. Alexander Bennet,* are due entirely to its effects upon the posterior column of the spinal cord; an observation that may make the drug useful in locomotor ataxia. He stated that he is now making clinical investigation in this direction.

This increased activity of the central nervous system is usually followed by a quiet, composed, self-satisfied condition of the mind and body that eventuate in sleep. These agreeable effects are accompanied with loss of appetite, frequently with nausea, constipation and diminished activity of the kidneys, of the sexual functions, and of the skin. In large doses, two to ten grains of the alkaloid, there are produced tinnitus aurium, photophobia, illusions, hallucinations, great loquacity, and a marked tendency of the mind to exaggeration and misrepresentation. If continued for some time this dose produces perversion of the affections, a disturbance of the moral emotions, a tendency to quarrel with friends and former associates, and to form alliances with persons formerly regarded as inferiors.

This state of the nervous system may become very like delirium tremens, with the same kind of muscular tremor and the same kind of horrible hallucinations. During this time the loss of appetite and diminished activity of assimilation re-

*British Medical Journal, April 18, 1874.

sult in extreme pallor of the face, dryness of the skin, extreme constipation, very much diminished urinary excretion, loss of sexual function, and great emaciation.

Second. *Cocaine in the Alcohol and Opium Inebrieties.*—Much has been written upon the use of this drug in efforts to cure this form of nerve mal-nutrition. Louis Baur, M. D.,* in an admirable article details his experience with it in a case of alcohol inebriety. He began with one-fifth of a grain, which the patient soon increased to ten grains by hypodermic injection, with the same disastrous result upon the nervous system as has been mentioned; but he expresses the opinion that cocaine inebriety was less objectionable than the alcoholic.

Dr. Erlenmeyer† gave it, in various doses, in 236 cases of opium inebriety, and expresses sentiments that entirely agree with PROFESSOR BROWER'S. He says that while cocaine does modify and mitigate the phenomena of opium-abstinence, its effect is only transient and of brief duration; he regards it of trifling value as a substitute for morphine.

Dr. J. T. Whittaker‡ reports, in an elaborate paper, the results from its hypodermic use in two cases of opium-inebriety that were satisfactory. Dr. Palmer, of Louisville, Ky., who was one of the first to advise its use in such cases, continues to be an enthusiastic advocate of the drug.

PROFESSOR BROWER'S experience is against its use in either of these inebrieties; it undoubtedly makes the withdrawal of either of these agents much easier for the patient, because its effects are so similar to opium and alcohol that he scarcely feels the need of either; but you place within his reach an agent much more rapidly disastrous and destructive to the

---

* Weekly Medical Review, Vol. 1, No. 12.

† Centralblatt fuer Nervenheilkund, July, 1885.

‡ Medical and Surgical Reporter, Aug. 15, 1885.

nutrition of the cerebral convolutions; an agent that will soon sink him to a degradation much lower than is possible with either of the others.

Third. *Cocaine in Melancholia.*—The best results yet obtained from the administration of the drug have been in conditions of mental depression. Dr. Jerome K. Bauduy,* in a valuable paper read before the American Neurological Society, June 17, 1885, relates a very extensive experience with the drug in melancholia. His method was to inject one grain of the muriate of cocaine, and he frequently witnessed the morose, silent, taciturn patient, a prey to the most profound grief or sadness, recover his normal self, begin to talk about his case and wonder how he could ever have experienced such gloomy ideas. He reports one case of suicidal melancholia which recovered in less than one month, and to whom he only gave five injections of cocaine. Dr. Alex. B. Shaw,† in an able paper on the uses of the drug, speaks with the same degree of positiveness of its value in the insanities with depression.

PROFESSOR BROWER'S own experience with cocaine in this form of insanity is in accord with Drs. Bauduy and Shaw. Although the bad effects of the drug upon the digestive and assimilative processes, and upon the secretions, have frequently disappointed him in its use. He has observed his valuable suggestion of giving the drug several hours before eating, in order to avoid the anorexia and nausea, but even with this precaution he has frequently found it impossible, while using it, to give that abundance of food, systematic feeding, which, after all, is the most valuable therapeutic measure in the relief of melancholia.

* New York Medical Journal, Sept. 26, 1885.

† The Weekly Medical Review, Vol. XII., No. 17.

Then again the excretory organs are often at fault; indeed, often the foundation of this form of insanity, the malnutrition of the brain being due to the accumulation in the blood of the waste product of tissue metamorphosis. In such cases the further depression added to those vital necessities by cocaine, must more than counteract the beneficial effects of cerebral stimulation. In such cases alteratives and stomachic tonics added to the treatment may make it successful.

He recalled two cases of profound melancholia. One a physician, aged 45, from a neighboring town of this State; an uncomplicated case, the result of excessive professional work in a large country practice. He received the cocaine in one grain doses three times a day, with pil. hydrargyri, aloes and strychnia. His recovery was rapid, and has continued for four months. The other case was that of a woman aged 48, from Indiana, laboring under melancholia, that seemed to have its origin in the fret and worry induced by a tumor of one of the mammary glands. Under this combined treatment recovered about as rapidly as the case detailed by Dr. Bauduy. In both these cases the drug was administered in pill form, and probably because of its combination did not interfere with the free use of egg-nog and other concentrated food in large quantities.

He has now under treatment a case of melancholia in which he is using the drug with the atomizer, using about four grains a day, on the nasal mucous membrane. The stimulating effects on the brain are manifested in a very few minutes after it is used. He is of the opinion that cocaine is the most valuable recent addition made to the therapeutics of melancholia, especially if its bad effects are guarded against in the way suggested.

Fourth. *Neurasthenia.*—Cocaine is of value in the treatment of this tedious and perplexing derangement of the nervous system. Dr. J. Leonard Corning,* in his scientific review of the cerebral form of this disease, calls it "the remedy *par excellence.*" Dr. William Oliver Moore,† in a very valuable paper on the physiological and therapeutical effects of coca and its alkaloid, gives his personal experience and the observations of others as to the value of the drug in all depressed conditions of the nerve centres, as well as its effects upon himself in various doses.

PROFESSOR BROWER'S experience coincides with the testimony of these writers, but he observes the same care in sustaining the digestive function and stimulating the eliminations as stated before. Cocaine, as mentioned in the beginning of this paper, is as powerful for evil as for good, and it requires no special prophetic gift to say that more disastrous results will be experienced by the laity from its indiscriminate use, than have been known from either opium or alcohol. Indeed, its action upon some persons in moderate doses is alarming. Dr. G. W. Kennicott‡ relates a case of poisoning from three and a third grains of the drug applied to the nasal mucous membrane, in the case of a female aged twenty-five, who had been using it for hay-fever. When he arrived she was in an alarmingly comatose condition, from which she recovered in about three hours, under the liberal use of brandy, ammonia and digitalis, with heat to her extremities and epigastrium.

DR. J. C. SPEAR,§ U. S. Navy, publishes a case of alarming coma closely simulating opium-poisoning, in a private, U. S.

* Brain Exhaustion.

† Quarterly Bulletin, New York Post Graduate School, Vol. I., No. 1.

‡ Chicago Medical Journal and Examiner, Oct., 1885.

§ The Medical Record, Nov. 14, 1885.

M. C., aged twenty-nine, the result of the hypodermic use of ten grains in divided doses, extending over about twelve hours. The case was supposed to be opium-poisoning, and was treated with atropia, coffee, and flagellation, and in about nine hours he recovered from the immediate effect of the poison. Dr. T. H. Burchard* gives an account of a case in which the hypodermic injection of four-fifths of a grain produced a sudden and complete loss of consciousness, and in which respiration stopped, and the radial pulse was scarcely perceptible. Artificial respiration, hypodermic injection of one-twelfth grain of atropia, and sinapisms to heart and extremities, relieved the patient. Fifteen minutes after the prostration the pulse was forty-eight and feeble, the respiration seven or eight, and the pupils contracted. Unconsciousness continued about twenty minutes.

DR. MERRIAM† relates the case of a gentleman who had been taking cocaine for four months for sick headache, beginning with about two grains a day, and gradually increasing till he was taking from ten to fifteen grains daily. He was very weak, with a pulse of 100, and his mind wandering somewhat as in delirium tremens. Drs. Bauduy and Shaw, in their papers already mentioned, dwell especially upon the dangers of the continued use of the drug in large doses. Professor Brower's experience is in accord with these several observers. Several cases of the poisonous effects of the drug have been under his treatment during the past six months, and he called the attention of the Society to two of these cases, both physicians:

First. The case of Dr. W., aged about thirty, of excellent

* The Medical Record, Dec. 5, 1885.

† Quoted by the Medical Record, Nov. 28, 1885, from Ohio Medical Journal.

physique, of neurotic tendency by inheritance, who began the use of the hydrochlorate of cocaine upon the nasal and pharyngeal mucous membranes for hay-fever. He gradually increased the dose to five grains taken in one dose in the evening, when his attack of hay-fever was usually most distressing. This dose gave almost immediate relief from the hay-fever, and gave a sense of mental stimulation very like champagne. He was almost at once seized with a desire for brain-work, and would pass the greater part of the night reading and writing on professional topics, experiencing a keenness of perception and a mental vigor greater than normal. He describes his sensations during the period of activity of the drug as exceedingly delightful. Towards morning he would fall asleep, and on the next day he would have no appetite and but little desire for work, the excessive stimulation having been followed by a corresponding depression of the vital forces.

He soon found a very irregular and rapid action of the heart, and passed by rapid stages to a condition of deplorable neurasthenia, from which he is slowly recovering. He had the same derangement of digestion, assimilation and elimination that have been already mentioned. He had but little desire for food, a thickly coated tongue, a feeble digestion, considerable emaciation, urine scanty, and much of the time loaded with uric acid and urates, with dyspnœa and the irregularity of heart action heretofore mentioned. The stools for some time were chalky, skin dry and pallid, pupils dilated, reflexes, especially the patellar tendon, much increased, and muscular powers much diminished.

He continued the use of the drug about ten days, and stopped it because he feared its enslaving power. This profound depression of the nervous system followed immediately upon its stoppage. The agents used to overcome this neurasthenia were an abundance of easily digested food, mild alteratives, moderate alcoholics, strychnia in small doses, cinchona, and the compound syrup of hypophosphites. As stated, the depression is gradually passing away, but the ten days' use of cocaine has incapacitated him for four months from the practice of his profession, and the probability is that at least three months more will be required to complete his restoration.

Second. The case of Dr. B., aged thirty-five, a man of decidedly neuropathic temperament, a hard-working, conscientious, and skillful physician, in the enjoyment of a fair but very laborious practice, with an excellent family history. Three years ago, with Professor Brower's assistance, he discontinued the use of opium, which he had been using excessively. He began the use of cocaine last May in one-eighth grain doses, having been led to believe it to be a harmless stimulant, and being at the time much run down by excessive professional work. It gave him such a sense of well-being as he had never experienced from any agent before, the sense of complete repose and self-satisfaction it produced being very much more marked and agreeable than that derived from opium. He gradually increased the dose until he consumed about fifteen grains a day by hypodermic injection. The large doses soon began to produce mental disturbance; he became irritable, quarrelsome, impetuous, and considered himself to be possessed of a mission, and that was to revolutionize the medical practice, claiming to be able to cure all diseases by the potency of cocaine. He gave it indiscriminately to all his patients. He gave it to obstetric cases and to syphilitic cases. He gave it to his wife, his three children and his mother. He was formerly a modest man of science; he became bold and unscientific in his method, went about engaging in lawsuits, carrying a pistol and frequently brandishing it in public places, threatening vengeance on all who dared to doubt the correctness of his various extravagant statements, a perfect terror in his neighborhood. He had been a very devout member of the Roman Catholic Church, but the priest of the church could now do nothing toward restraining his wild impetuosity. He neglected his practice, and by his manner alienated those whom he did not neglect, so that very soon he lost it. Piece by piece his horse, his buggy, and his furniture disappeared, until his family was reduced to poverty. Repeated efforts to persuade him to stop the use of the drug were unsuccessful; indeed, simply resulted in making Professor Brower the recipient of his wildest denunciations and of his severest threatenings. Several physicians and druggists who made attempts to restrain him met with an equally positive rebuff. The same general deterioration as before noticed, was manifest in his case, extreme pallor and dryness of the skin, great emaciation, loss of appetite and no desire for sleep, so that for at least one week he did not assume the recumbent position.

He continued to go from bad to worse until his friends thought it best to restrain him. In pursuance of this object Professors F. L. Wadsworth and Brower appeared before the County Court and advised his removal to the Washingtonian Home. Here the cocaine was gradually withdrawn, but his mental extravagance continued unabated. He left this institution clandestinely, and is now supposed to be in Canada.

To sum up, he said:

1. Cocaine in small or moderate doses is a cerebral stimulant, but produces derangement of the digestive and assimilative functions, and diminishes the elimination of waste.

2. The use of cocaine in the alcoholic and opium inebriates is not satisfactory; while it is a more or less perfect substitute, yet its use is attended with greater danger than alcohol or opium.

3. The use of cocaine in mental depression, if we carefully guard against the depressing effects of the drug upon digestion and assimilation, will often give better results than any drug hitherto used.

4. The use of cocaine in neurasthenia is a valuable addition to the treatment.

5. The drug, if administered in large doses persistently, causes a very marked deterioration of the central nervous system, producing a profound cerebral neurasthenia, and may produce such a mal-nutrition of the cerebrum as to develop insanity.

6. Cocaine, occasionally, in doses heretofore regarded as small, produces alarming depression of the central nervous system.

PROFESSOR E. L. HOLMES opened the discussion by saying that the South American Indians eat large quantities of the coca leaf, without injury.

PROFESSOR BROWER said that in the early history of Peru the Catholic Church authorities sought to stop the use of coca in that way because of its deleterious effects upon the people. But it was impossible to do it, they used it clandestinely, and the prohibition was withdrawn. He said that used in that way the result was a similar condition of mental deterioration as that mentioned in the paper; while these people were capable, under its influence, of performing muscular efforts in climbing the hills and mountains of that country, they were a puny, sallow, emaciated people, with intellectual capacity very little above the brute creation.

PROFESSOR HOLMES said that his experience had been wholly in connection with local applications on the eye. He had seen one case, in which he had placed cocaine in both eyes for an operation, in which it was followed by considerable depression and nausea through the night, but no alarming symptoms. He had twice used it in his office for strabismus, placing a not unusual quantity in the eye, where the patient felt sick and almost slipped out of the chair, and the operation was performed with the patient lying on the floor, but he suspected it to be a fit of fainting at the sight of the instruments. He had found from experience that very minute doses will be absorbed, but had never seen the slightest influence from the amount which is usually given in the eye. It is exceedingly satisfactory in performing operations, and in removing little motes and pieces of iron or steel lodged in the cornea.

PROFESSOR C. W. EARLE said it appeared to him that when Dr. B. was in the Washingtonian Home he got along with a very small amount of the drug, very much less than he had anticipated it would be possible for him to get along with. That Dr. B. informed him that he had been in the habit of taking from 15 to 18 grains a day. After he went into the Home, the first three days he took less than three grains, and after that time did not take any. He regained his appetite very well, and appeared to improve in every respect up to the time that his wife and brother or brother-in-law came from Canada to visit him, then he was seized with an idea that he must go home, or at least to Canada, and from that time he was uneasy and did not do well. Professor Earle said that it seemed to him that there had been more said in regard to the use of cocaine than there was any use in saying. While Dr. B. was at the Home he was not unlike an ordinary opium-eater. The depression which followed withdrawal was somewhat similar, although there were none of the symptoms of dizziness, or nausea and sneezing, which are usually present after the withdrawal of opium.

PROFESSOR SARAH HACKETT STEVENSON said that she was surprised at the deleterious effects found by Dr. Brower from the use of cocaine in hay-fever. She had had a number of cases in which it was used, without any of the symptoms mentioned in the paper. She used a 4 per cent. solution, applying with a camel's-hair brush, or simply by snuffing. Professor Stevenson said she had used cocaine a great deal hypodermically in surgical operations with good effect so far as anæsthesia was concerned.

DR. W. F. COLEMAN said there are two lines of practice in treating the narcotic habit: to cut a man off from his drug completely, at once, or to taper him off. He believed in depriving him of the drug at once. If he found a man being poisoned he would not add another dose, but stop it immediately. He knew nothing practically of the internal use of cocaine, but was surprised at the grave effect mentioned by Dr. Brower. Cohn-Mueller asserts that he kills a frog with four-tenths of a grain, but to human patients he gave indiscriminately 5 grains and it produced no perceptible ill-effect. Locally, Dr. Coleman had had some experience with the drug, and there is this point about its immediate effect, it relieves pain in acute otitis, but it is a grave question whether it does not prolong the case, and while it relieves pain it is doubtful if it is better than hot water or other remedies. In its application to the eye there was not this objection, it produces anæsthesia and allows almost any operation to be performed without pain; it does not prolong congestion, and gives permanent relief.

PROFESSOR D. W. GRAHAM said that he noticed Dr. Brower quoted a case reported by Dr. Burchard in the *Medical Record*, in which a patient sustained loss of consciousness and stoppage of the pulse after an injection of four-fifths of a grain of cocaine. Professor Graham doubted very much whether the bad effects were due to cocaine; we get the same effect in many patients by injecting water, or by showing them the hypodermic syringe, and he thought it probable that it was simply a fainting-fit. There was nothing in the report that would lead to any other conclusion except that Dr. Burchard says it was the result of the injection of cocaine. Professor Graham doubted the conclusion and thought it unreliable.

DR. F. M. WELLER said he had had some experience in the use both of cocaine and the fluid extract of coca. He had never used such large doses as those mentioned, but had seen that very large doses of the drug would produce unpleasant symptoms about the head, dull heavy headache, something like doses of other narcotics. Hypodermically he had used as much as five grains at one time without any unpleasant effects whatever. He was inclined to think that the real difficulty in some of these cases was that some other circumstance was overlooked. It seemed to him that some idiosyncrasy might exist that would make one peculiarly susceptible to this kind of narcotic, and it would hardly be reasonable to charge it all to cocaine. His reading of the history of the use of the drug in South America, where it originated, led him to think that it could be used a long time without injury. He thought one lesson is to be learned from the paper, viz., that no drug should be continued beyond the time of its necessity. That principle laid down and strictly adhered to, the patient would never suffer from the deleterious use of any drug.

PROFESSOR BROWER in closing the discussion, said that Dr. B. bought cocaine wherever he could get it, and he did not know what preparation he used. The other physician mentioned in the paper used Merck's cocaine altogether. He used it for hay-fever and took five grains by inhalation every day for ten days. Professor Brower said that he did not suppose that such disastrous effects as occurred in these cases and others, would result unless there was some weakness of the nervous system; he thought possibly a person perfectly robust and with a well-governed nervous organization could take cocaine with impunity, but a person with a nervous temperament could not use this drug continuously without some such results following. He said his plan of treatment was that of gradual withdrawal, as well with opium and chloral as with cocaine. This plan could be followed with less inconvenience to the patient. He said he was well aware that cocaine was one of the most valuable additions to the therapeutics of hay-fever, it had been administered in perhaps thousands of cases in Chicago, and with few disastrous effects. As to the case reported by Dr. Burchard, it was reported as a case resulting from hypodermic injection, and it struck Professor Brower as being a reasonable result in a very susceptible individual, but it might be that the man had a fainting-fit. The case was reported in the *Medical Record* of Dec. 5, 1885; it impressed Professor Brower as being undoubtedly a case of cocaine-poisoning.

After the reading of the paper entitled "Opium Smoking," by Professor C. W. Earle, and one entitled "Hygienic Clothing" by Dr. L. L. McArthur, on which there was no discussion, the society adjourned.

